# 

**Published:** 1858-03

**Authors:** 


### CONTENTS OF THE MARCH NUMBER.

### ORIGINAL COMMUNICATIONS.

| | Page |
|---|---|
| ART. I.—Fractures of the Ribs, and of their Cartilages. By Frank H. Hamilton, M. D., | 578 |
| ART. II.—Reduction of Dislocations of the Femur by Manipulation. By F. H. Hamilton, M. D., | 581 |
| ART. III.—Albuminuria, Independent of Organic Disease of the Kidneys. By James M. Newman, M. D., | 5[illegible]9 |
| ART. IV.—Reports of Cases occurring in the Medical Ward of the Buffalo Hospital. Reported by Dr. A. W. Nichols, | 608 |
| ART. V.—The Principles and Practice of Obstetrics, | 616 |
| ART. VI.—Monthly Record of Deaths in the City of Buffalo. By H D. Garvin, M. D., Health Physician, | 619 |

### EDITORIAL DEPARTMENT.

| | |
|---|---|
| The Supra Renal Capsules, | 621 |
| Annual Meeting of the Erie Co. Medical Society, | 628 |
| Death of Morgan G. Lewis, M. D., | 631 |
| The Sale of Poisons. | 634 |
| The Sydenham Society, | 635 |
| New Stand for the Compound Microscope, | 637 |

